# Explaining the stability of cooperation in agricultural industry chains based on the institutional theory: Multiple mediating effects of perceived value and trust

**DOI:** 10.3389/fpsyg.2022.1094879

**Published:** 2023-01-12

**Authors:** Rong-Rong Zhao, Qiao Wang, Yuan Tian, Qiu-Hua Chen

**Affiliations:** ^1^College of Economics and Management, Fujian Agriculture and Forestry University, Fuzhou, China; ^2^Research Center for Ecological Civilization, Fujian Provincial Social Science Research Base, Fuzhou, China

**Keywords:** agricultural industry chain, institutional theory, composite multiple mediating effect, perceived value, trust, cooperation stability

## Abstract

**Introduction:**

Due to the superposition of multiple complex socioeconomic environments and the complexity and uncertainty of the agricultural industry chain itself, the agricultural industry chain has become unstable, jeopardizing its long-term sustainability.

**Methods:**

The purpose of this study is to construct and validate a stability mechanism model of cooperative relationships within agricultural industry chains based on the institutional theory. The questionnaire survey method was used for empirical analysis.

**Results:**

The results show that imitative pressure, mandatory pressure, and normative pressure have significant positive effects on the stability of cooperative relationships in agricultural industrial chains. Besides, perceived benefits, perceived risks, and trust play composite multiple mediating roles between imitative pressure and cooperation stability, and between normative pressure and cooperation stability in agricultural industrial chains. Perceived benefits and trust play partial mediating roles in the stability of cooperative relationships between mandatory pressure and agricultural industrial chains.

**Discussion:**

This study is conducive to further understanding the cooperative psychology of agricultural industry chain operators. And this research can provide a reference for managers to take targeted measures to deal with the instability in the development of agricultural industry chains.

## 1. Introduction

Since the superposition of multiple complex socioeconomic environments, agricultural industry development also faces increasing variability, discontinuity, and uncertainty (Kou et al., 2016; He and Yang, 2021). On one hand, under the impact of COVID-19, global climate change challenges, and economic downward pressure, the agricultural industry chain faces challenges such as upstream and downstream supply and demand mismatches, as well as risk transmission, and is prone to the risk of chain breakage when it is disturbed by external risks (Liu and Ma, 2020; Morton, 2020; Zhao and Yang, 2020). On the other hand, in order to promote the development of agricultural modernization, it is necessary to further deepen the coordinated development of rural primary, secondary and tertiary industries, and build a new driving force for agricultural development with industrial integration (Guan, 2018). The key to solving these problems is to cultivate a stable agricultural industry chain. The core of cultivating an industry chain is to establish stable industrial linkages among industries, i.e., to realize the linkage and synergy effects through the linkage development of primary, secondary, and tertiary industries, to reduce transaction costs, enhance agricultural risk resistance, and increase the comparative benefits of agricultural products, thereby achieving agricultural development and increasing farmers’ income (Xiao, 2012). In the agricultural industry chain, several links are closely related to the provision of agricultural products, including pre-production, production, post-production, circulation, and marketing of agricultural products. As a complex system with multiple links and stakeholders, the system interacts with each other in different ways and is affected by changes in the internal and external environment as well as unstable risks (Guo et al., 2013; Cheng et al., 2019; Yu and Zhang, 2019). In order to achieve the synergy of the agricultural industry chain so as to cope with external risks as well as the need for internal sustainable development, it is essential to promote the stability of cooperative relationships among different operating subjects in the agricultural industry chain.

Multiple economic agents are distributed in the agricultural industry chain, and each agent is linked to an organic economic system through the same industrial base and market (Qin, 2022). This economic system is generally based on contractual linkages that determine the existence of contractual risk in the agricultural industry chain (Zhou and Long, 2019). Operating agents in different positions of a chain hold asymmetry market information, resulting in different bargaining power in the chain. It extremely difficult to avoid moral risk caused by opportunistic behavior and adverse selection by the parties to the transaction, even in the presence of contractual restrictions (Simangunsong et al., 2016; Xu, 2018). However, in real practice, even if there are conditions for opportunistic behavior, the agricultural industry chain still maintains stable cooperation, and the industrial operators at different links have a strong trust relationship. This process does not solely involve institutional pressures such as the contract. Accordingly, in some cases, even if the conditions of default risk do not exist, it is difficult for the operating subjects in the industry chain to form stable cooperative relationships. In addition, it has been found that the role of institutions is weakened under the crisis of trust. In asymmetrical information or high-risk environments, organizations are pressured to make industry-integrated and policy-compliant decisions to gain stakeholder’s support and acceptance (Meyer and Rowan, 1977). The industrial operators in the agricultural industry chain are thoughtful individuals or groups. The mechanism of driving the choice of a stable cooperative strategy by the internal psychology of industrial operators under institutional pressure is not yet clear. Therefore, this research uses the institutional theory to explore the psychological mechanism of the stable cooperative decision-making process of agricultural industry chain operators and identify the key points in the selection of stable cooperation among agricultural industry chain operators. The purpose is to better address the instability in the development of agricultural industry chains and promote in-depth integration.

Based on the institutional theory, the questionnaire survey method was used to investigate the agricultural industry chain operators. First, the functions of the perceived value and trust of agricultural industry chain operators under institutional pressure are analyzed. Second, the relationship and path of institutional pressure, perceived value, and trust to maintain the stability of the agricultural industry chain cooperation relationship are studied. Moreover, this study expands the related research to the explanation of organizational psychology and behavior by institutional theory, and facilitates further insight into the psychological changes of organizations and individuals in agricultural industry chains. This not only helps to address the instability of agricultural industry chain cooperation, but in turn provides a reference for promoting the stability and modernization of agricultural industry chains.

## 2. Theoretical basis and research hypotheses

### 2.1. Relationships in the agricultural industry chain

The agricultural industry chain is a complex system involving multiple and multi-level operators, which often works together within specific industry sectors (Bryceson and Smith, 2008). Originally small-scale independent operators in agricultural chains are closely integrated into interdependent players based on the production and distribution of value chains (Boehlje, 1999). Agricultural industry chains link the producers of agricultural products to markets, thereby expanding the supply capacity of agricultural markets, and different operators in the chain form special value relationships based on partnership (Mcmichael, 2013). Cooperation in the chain is subordinated to price relations, and it converts agricultural resources into values. These values are transformed into profits and are then redistributed along the value chain among processors, retailers, distributors, and traders. In industrial chains, contracts are an important embodiment of vertical collaboration and close relationships between agricultural chain operators (Banterle and Stranieri, 2008). On the one hand, contract-based agricultural industry chain cooperation allows farmers to share more profits (Maertens and Velde, 2017). On the other hand, institutional innovations based on cooperation, such as contract farming, can help to reduce transaction costs and risks for smallholders. By linking smallholders with processors and retailers in the chain, access is available to additional financial capital from banks, technologies can be obtained, and extension and buy-back arrangements and monitoring of food security can be realized (Mcmichael, 2013). However, agricultural industry chain cooperation is not always successful, it is influenced by multiple factors such as trust, commitment, and transparency between chain partners (Bhagat and Dhar, 2011).

### 2.2. Institutional theory

The institutional theory gives the reason why organizations within the same field often behave and look the same (DiMaggio and Powell, 1983). The institutional theory states that organizational structures and processes tend to acquire meaning and stabilize themselves, rather than based on expected efficiency and effectiveness. According to institutional theory, laws, rules, and beliefs in the external environment constrain the structure and behavior of organizations (Meyer and Rowan, 1977; DiMaggio and Powell, 1983; Chen and Yao, 2015). Organizations within the same institutional domain may gradually converge due to the constraining pressure of institutions (Sun and Wang, 2022). The isomorphic pressure exerted by institutions on organizations can be divided into three types: mandatory pressure, imitative pressure, and normative pressure (Li et al., 2022). Mandatory pressure is the formal and informal pressures exerted on an organization by resource entities upon which the organization depends (Teo et al., 2003). Imitative pressures arise from uncertainty and generated by organizations coping with this uncertainty by imitating the behavior of other organizations (Jiao et al., 2021a). Normative pressure refers to the pressure stemming from norms set by institutions such as professional or trade associations (Krell et al., 2016). Institutional theory suggests that institutions influence the behavior at three levels: individual, organizational, and inter-organizational (Oliver, 1997). The institution influence can be seen at the individual level where managers adhere to norms, habits, and customs. In the organization, it can be seen in uniform activities supported by a common political, social, and cultural framework. And at the inter-organizational level, it can be seen in the accepted organizational behavior by government and industry associations to promote society’s common values.

The purpose of cooperation is to achieve greater benefits for oneself and form alliances, and unified action between participants is one of the basic means of cooperation (Hu, 2018). Institutional theory is an important theory to explain the unity of organization members’ actions, and it can equally explain the unity of cooperative action. Industrial chains are strategic alliances formed by different sectors in the chain, and are the product of alliance and cooperation among multiple industrial operators. Therefore, constraints by institutions are of necessities to ensure the stable industrial chain. The formation of cooperative relationships in the agricultural industrial chain is also the result of institutional isomorphism. Similarly, the subject members in the industrial chain system are influenced by institutions when choosing stable cooperative strategies. For example, the institutional pressure of contract formation in chain cooperation promotes the process of vertical association of different operators. Therefore, this study strats from the perspectives of mandatory pressure, imitative pressure, and normative pressure, in order to examine how institutional pressure affects the stability of cooperative relationships among agricultural industry chain operators.

### 2.3. Perceived value theory

As part of consumer behavior research, perceived benefits and perceived risks are derived from the perceived value theory, which can be used for consumers to compare and evaluate the perceived benefits and perceived risks of a product or service. Thus, consumer behavior can be predicted based on perceived value (Li et al., 2021). An individual’s perceived benefit is the subjective perception of the possible positive results, while the perceived risk is the subjective perception of possible negative consequences (Zeithaml, 1988). The theory was applied to several other fields to explain the behavior of actors. It was found that there is a positive relationship between perceived benefits and behavior, and higher perceived benefits are associated with greater behavior intention (Li et al., 2020; Wang and Yan, 2022). In contrast, there is a negative relationship between perceived risk and behavior (Krell et al., 2016). When the perceived risk is equal to or even exceeds the subject’s tolerance range, the likelihood of behavior intention is decreased (He et al., 2014). The agricultural value chain involved in the agricultural industry chain is a relationship between value addition, value distribution, and risk sharing in the chain. Cooperation can reduce transaction costs and reduce speculative risks, in addition to other risks that may arise (Mcmichael, 2013). When choosing stable cooperation, both the perceived benefits and perceived risks should be considered. When the stable cooperative behavior is perceived to bring relevant benefits, it will be adopted; otherwise, non-cooperative strategies will be choosed. For example, member companies in the agricultural industry chain may face increased input costs when they choose a cooperative strategy, however, the operation will be ended when the costs outweigh the benefits. In addition, there maybe also many uncertainties that negatively affect the cooperative behavior of the members of an agricultural industry chain, need to be further studied.

### 2.4. Model construction and research hypothesis

**FIGURE 1:**
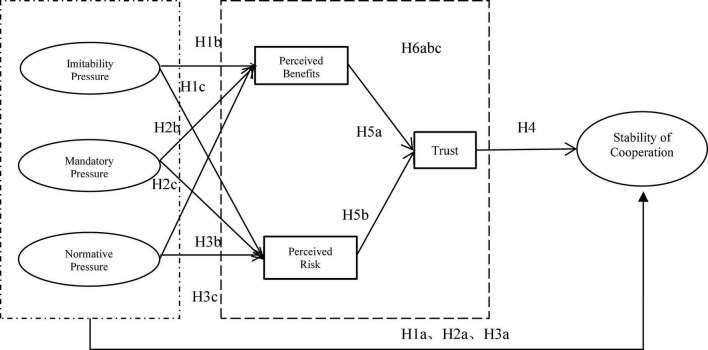
Schematic diagram of the research hypothesis model.

An agricultural industry chain consists of strategic alliances formed for cooperation and income generation. The development of such an alliance must depend on a particular system of cooperation (Long and Liu, 2008). According to the institutional theory, the cooperative system of the agricultural industry chain can be categorized into three types of isomorphic pressures that drive the chain members to adopt cooperative behaviors at different nodes of the chain, i.e., mandatory, imitative, and normative pressures. However, it is not difficult to find out from the speculative behavior among various entities in the industrial chain in reality that it is challenging to maintain a long-term stable cooperation between different business subjects in the industrial chain only by the system. In the process of socio-economic development, whether individuals or groups trust in others is an important silent factor in the efficiency of complex social organizations, and trust is an essential component of any social group’s effectiveness, adjustment, survival, and development (Rotter, 1967; Lewis and Weigert, 1985; Fukuyama, 1996). Trust can further explain the mechanisms of the stability of cooperative relationships in the agricultural industry chain. In addition, according to the perceived value theory, individuals or organizations make a comprehensive assessment of the possible benefits and risks of behavior before taking any action, and use the assessment results to make choices (Zeithaml, 1988). Based on this, this study constructs a conceptual model based on the institutional theory to explain the inherent mechanism of the stability of cooperative relationships in agricultural industry chains, as shown in Figure 1.

#### 2.4.1. Institutional pressure, perceived value, and cooperation stability

Imitative pressure refers to coping with uncertainties by imitating the behavior of other individuals or enterprises (Sun and Wang, 2022). Industrial integration is a complex process that brings uncertainty to individuals, enterprises, and organizations. In the agricultural industry chain, industrial operators tend to imitate the successful decision-making behaviors of subjects similar to themselves in the organization. Many organizations or individuals are involved in agricultural industry chains, such as multiple farmers. When some farmers adopt the stable cooperative behavior and achieve higher returns, others will follow them and also choose the stable cooperation. There are several advantages of the imitative behavior, such as reducing search costs (Lin et al., 2019), reducing experiment costs (Levitt and March, 1988), and avoiding the risk as the first mover (Lieberman and Montgomery, 1988). Industrial operators in the chain will gain more information and experience from the behavior of organizations or individuals in the same chain who are on equal footing, avoiding risks and stabilizing growth after the cooperation or default. Therefore, hypotheses 1a, 1b, and 1c are proposed.

Hypothesis 1a: Imitative pressure has a positive effect on the stability of cooperative relationships in the agricultural industry chain;

Hypothesis 1b: Imitative pressure has a positive effect on the perceived benefits of cooperative relationships stability in the agricultural industry chain;

Hypothesis 1c: Imitative pressure has a negative effect on the perceived risk of cooperative relationship stability in the agricultural industry chain.

Mandatory pressure originates from dominant agents on which the organization relies, which may control important resources or maybe regulatory bodies with power. The dominant agent requires the organization to adopt a program or organizational structure that meets its interest (Lin et al., 2019). By imposing mandatory pressure on weaker subjects, either formally or informally (DiMaggio and Powell, 1983), the strong dominant can transform power into authority by restricting, regulating, or adjusting their behavior (Scott, 1987). Through various institutional forms, the strong organization in a coalition organization will constrain other members to accept values or logic of action in line with their interests, thus creating an environment conducive to the organization’s development as a whole (Peng et al., 2018). It is common for the agricultural industry chain to have a leading unit, such as a leading enterprise. The leading enterprise or another dominant unit sets the rules and programs for cooperation. Under the influence of mandatory pressure, the operating agents in the chain, especially the non-dominant operating agents, decide whether to adopt the cooperation strategy by weighing the benefits and analyzing the concept of compliance with the cooperation rules and precautions. Complying with the dominant operator’s requirements and instructions can result in continuous access to resources or smooth channels, which positively influences the cooperation. Based on this, hypotheses 2a, 2b, and 2c are proposed in this study.

Hypothesis 2a: Mandatory pressure has a positive effect on the stability of cooperative relationships in the agricultural industry chain;

Hypothesis 2b: Mandatory pressure has a positive effect on the perceived benefits of cooperative relationships stability in the agricultural industry chain;

Hypothesis 2c: Mandatory pressure has a negative effect on the perceived risk of cooperative relationships stability in the agricultural industry chain.

An organization’s normative pressure is primarily derived from its members’ collective expectations, expressed as values and norms, and serves as a foundation for the maintenance of collective action (DiMaggio and Powell, 1983). The members of social groups also maintain their legitimacy in the organization by adhering to the goals and behaviors of the organization (Dowling and Pfeffer, 1975). In reality, most normative pressure comes from institutions such as professional or trade associations (Krell et al., 2016). However, normative pressures do not rewards or sanction organizations directly based on whether they behave in accordance with norms as mandatory pressures do. The sharing and dissemination of these norms by profession members through relevant channels can help negotiate the agreement and thus increase the influence of these norms on organizational behavior (Teo et al., 2003; Yao and Li, 2022). To publicize the importance of integrating the three rural industries, the government and social forces have established various agricultural-related websites, and the advancement of information technology also encourages industry chain members to adopt consistent and cooperative behaviors. In addition, agricultural business subjects accept normative pressure from the system to maintain their long-term legal status. Based on this, hypotheses 3a, 3b, and 3c are proposed in this study.

Hypothesis 3a: Normative pressure has a positive effect on the stability of cooperative relationships in the agricultural industry chain;

Hypothesis 3b: Normative pressure has a positive effect on the perceived benefits of cooperative relationships stability in the agricultural industry chain;

Hypothesis 3c: Normative pressure has a negative effect on the perceived risk of cooperative relationships stability in the agricultural industry chain.

#### 2.4.2. Perceived value, trust, and cooperation stability

When an individual chooses to trust in a person or organization, it means that he/she will take risks from other person, and trust is a necessity in environments, relationships, and scenarios where risk exists (Baer et al., 2022). Nevertheless, taking risk does not necessarily imply trust, and sometimes it may only serve a common purpose (Zhang, 2020). Trust is a dynamic process, and the choice of trust is intuitively reflected in the corresponding trust behavior (Yu et al., 2022). In addition to being a complex adaptive system, the agricultural industry chain also requires trust, which is essential for the development of an organization (Shang and Chen, 2022). According to Jiao et al. (2021a), trust is a necessary condition for cooperative economic behavior. Considering the complexity and uncertainty of society, a high level of trust is required to support the cooperative behavior, and cooperation cannot exist without trust (Qu, 2018). Social exchange theory suggests that individuals and groups establish interactions in the context of exchanging with social characteristics, through which they exchange resources and receive valuable rewards or benefits. To maintain this social contractual relationship, trust must be generated and maintained (John, 1992; Li, 2014). It is possible to reduce uncertainty and risk in the transaction process and reduce transaction costs through trust (Lu et al., 2022). The degree of trust between collaborators also influences the effectiveness of cooperation (Xue et al., 2022). Sieczko et al. (2021) investigated the collective enterprises in rural areas and found that trust can promote cooperative relationships. In a complex cooperative alliance, the trust relationship shared by chain members also contributes to the stability of cooperation in agricultural industry chains. Therefore, hypothesis 4 is proposed in this study.

Hypothesis 4: Trust has a positive effect on the stability of cooperative relationships in the agricultural industry chain.

There are some conflicting views regarding the relationship between perceived benefits, perceived risks, and trust, with the main conflict as whether perceived benefits and perceived risks are antecedents or consequences of trust. Guo et al. (2021) argued that rural community residents’ perceptions or evaluations of the rewards of participating in rural tourism actions are important factors affecting their ability to build trust or maintain interactions with other actors. Therefore, perceived benefits are antecedents of trust. However, Wang and Yan (2022) argued that perceived benefits are a consequence of trust and that farmers are able to trust in others and thus have positive perceived benefit evaluations of the actions taken. The relationship between perceived risk and trust has also been found to be inconsistent. According to Kim and Prabhakar (2004), perceived risk and trust are juxtaposed and jointly influence customer behavior, although there is no clear causal relationship between them. Yan (2022) believed that the stronger the consumer trust, the weaker the corresponding perceived risk, which in turn facilitates purchasing behavior. The results of the empirical test by Zhuang et al. (2018) verified the conclusion that perceived benefits and perceived costs negatively affect trust and trust is a result of perceived value. In some studies, trust is considered as a moderating variable between perceived risk and behavior (Tao et al., 2022). However, trust is defined as the psychological belief that other person will not harm oneself and is willing to bear the harm from other person (Rousseau et al., 1998). Trust is built upon uncertainty and risk, and individuals and organizations evaluate and feel risk before building trust; therefore, this study hypothesizes that perceived benefits and perceived risks are antecedents of trust. Based on this, hypotheses 5a and 5b are proposed.

Hypothesis 5a: Perceived benefits have a positive effect on trust;

Hypothesis 5b: Perceived risk has a negative effect on trust.

#### 2.4.3. Composite multiple mediating effects of perceived benefits, perceived risks, and trust

The reality is often more complex, with institutional pressure, perceived value, and trust jointly influencing the behavioral decisions of agricultural industry chain operators. In the practice of agricultural production and operation, sometimes the real return of farmers cooperating with enterprises is lower than market prices, but this cooperation can also be maintained. Farmers have confidence in the enterprises and believe they will maintain the cooperation and pursue long-term revenue stability. Furthermore, the existence of rural social networks makes farmers more likely to trust in their friends and relatives, even if the cooperative returns are lower than those of unfamiliar objects. Hobbs (2020) pointed out that a stable supply chain partnership based on trust is the key to maintaining a stable supply chain and responding flexibly to external conflicts such as the Newcastle pneumonia outbreak. To analyze the stability of cooperative relationships in the agricultural industry chain, this study also explores how trust contributes to the stability of cooperative relationships in the agricultural industry chain from the perspective of trust, in addition to institutional and economic benefits. Based on these findings, the institutional pressure, perceived value, trust, and the stability of cooperative relationships are combined together in this study for analysis. The basis for studying the stability of cooperative relationships in agricultural industry chains is that agricultural industry chains are at risk of changing dynamics; the existence of the agricultural industry chain will first affect the perceived value of industrial operators on the entire business environment, and then affect the trust relationship between operators through the perceived value, and finally affect the stability of cooperative relationships in agricultural industry chains. Therefore, hypotheses 6a, 6b, and 6c are proposed.

Hypothesis 6a: Perceived benefits, perceived risks, and trust play composite multiple mediating roles between imitative pressure and cooperative relationships stability;

Hypothesis 6b: Perceived benefits, perceived risks, and trust play composite multiple mediating roles between mandatory pressures and cooperative relationships stability;

Hypothesis 6c: Perceived benefits, perceived risks, and trust play composite multiple mediating roles between normative pressure and cooperative relationships stability.

## 3. Research design

### 3.1. Measurement tools

Based on published literature, the scales of the key variables in this study are determined to analyze the characteristics of the agricultural industry chain. In order to measure the validity of the scales, questionnaires were distributed to agricultural industry chain operators, teachers, and graduate students in agriculture and forestry economics and management. The expression of the questionnaire was modified according to their suggestions. Finally, the scale of this study was obtained.

The scale of institutional pressure was divided into imitative pressure, mandatory pressure, and normative pressure, which was mainly drawn based on the scales of Liang et al. (2007) and Lin et al. (2019). Imitative pressure has four items, and the sample item is “Others in your chain have gained more through stable cooperation”; mandatory pressure has four items, with the sample item as “You have been punished for adopting a non-stable cooperation strategy”; normative pressure includes four items, with the sample item as “My upstream industry players adopt stable cooperative behavior.”

The scales for the perceived benefits and perceived risks sections were modified based on the reports by Kurnia et al. (2015) and Siamagka et al. (2015), respectively, to analyze the characteristics of industrial agents in the agricultural industry chain. The perceived benefits include five items, and the sample item is “Adopting a stable cooperative strategy meets my own development needs.” The perceived risks include four items, and the sample item is “Adopting a stable cooperative strategy requires a large investment.”

The scale of cooperative relationship stability was modified based on the scales of Li and Zhang (2007), Zheng (2009), Du et al. (2012), Wang et al. (2019) on the stability of cooperative relationships in supply chains. Combined with the concept of stability in agricultural industry chains, the scales were adjusted in terms of cooperation time, degree of shared information, cooperation expectations, communication, and cooperation response risk. After several adjustments, the scale of this study was set up, containing seven questions.

The scale of trust adapted the scale by Wang (2015) and Liu et al. (2009), including six items. The sample item is “I believe that the partner in the agricultural industry chain will timely accomplish what they have promised.”

The scale adopted a five-point Likert scale. Questionnaires were distributed to a small number of agricultural industry chain operators, graduate students and teachers in agriculture and forestry economics. Certain modifications on the questionnaire were made according to their suggestions.

### 3.2. Data collection

This study used the questionnaire survey method to collect data, and the questionnaires were distributed to different business subjects in the agricultural industry chain, including farmers, family farmers, cooperatives, personnel involved in agricultural product processing enterprises, and marketing personnel. The samples were collected from two main sources. First, the survey was completed online by sending the link of the Questionnaire Star questionnaires to appropriate research subjects; second, it was compiled based on the new vocational farmer training course offered by Fujian Agriculture and Forestry University, and interviews were then conducted. The survey was performed in two steps. First, it was a pre-survey, and 103 questionnaires were collected. Second, it was the official research, and 381 questionnaires were collected. The invalid questionnaires were removed, including those finish in a too short time, select the same answer for all options, and do not cooperate with other industrial operators. A total of 275 questionnaires were collected, of which 81 were completed offline through the New Professional Farmer Training Course. Drawing on Armstrong and Overton’s (1977) methodology, independent samples *t*-tests were carried out on the 81 offline interview questionnaires and 194 direct online questionnaires. The results indicated that there was no significant difference between the two datasets, suggesting no response bias. After collecting the questionnaires, the data were statistically analyzed using SPSS statistics 26 and AMOS 25.

There are 67.0% of males and 33.0% of females in the samples. A total of 53.6% of the respondents were aged between 22 and 44. In terms of education levels, high school or secondary school, college or bachelor’s degree are the majority, accounting for 33.3 and 45.3%, respectively. According to the distribution of business links, the majority of research subjects are engaged in agricultural product breeding, followed by sales. The agricultural products processing sector has fewer operators, accounting for only 17.8% of the total. In terms of business type, there are more family farms and cooperatives.

## 4. Data and empirical analysis results

### 4.1. Reliability and validity analysis

It should be noted that since the Cronbach’s α value of each variable in the model is greater than 0.7, KMO = 0.926 > 0.7, and *P* = 0.000 < 0.05 for Bartlett’s sphere test, this study has a high degree of reliability.

On this basis, the validity of the questionnaire was analyzed by validation factor analysis, mainly from three aspects: structural validity, convergent validity, and discriminant validity.

The structural validity results showed that the scale met the basic requirements for fitness (2/df = 1.592 < 3, RMSEA = 0.046 < 0.05, GFI = 0.858 > 0.8, IFI = 0.954 > 0.9, CFI = 0.858 > 0.8). From Table 1, almost all the latent variable question terms have factor loadings exceeding 0.7, few have loadings less than 0.7 but close to 0.7, and each variable meets CR > 0.7 and AVE > 0.5, suggesting that the sample measurement model is convergent. From Table 2, the maximum correlation coefficients of all latent variables are less than the square root of AVE, indicating good discriminant validity of the measurement model. This indicates that the scales are applicable to measure the research variables from the tested hypotheses.

**TABLE 1:** Convergent validity analysis of the evaluation model of cooperation stability in the agricultural industry chain.

		Paths	Estimate	AVE	CR
IP1	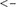	Imitative pressure	0.887	0.6819	0.8953
IP2	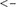	Imitative pressure	0.826		
IP3	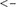	Imitative pressure	0.771		
IP4	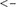	Imitative pressure	0.815		
MP5	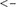	Mandatory pressure	0.914	0.6093	0.8595
MP6	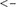	Mandatory pressure	0.843		
MP7	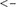	Mandatory pressure	0.678		
MP8	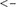	Mandatory pressure	0.657		
NP9	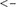	Normative pressure	0.939	0.6717	0.8903
NP10	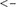	Normative pressure	0.812		
NP11	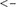	Normative pressure	0.779		
NP12	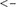	Normative pressure	0.734		
PB13	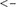	Perceived benefits	0.804	0.6073	0.8854
PB14	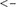	Perceived benefits	0.807		
PB15	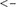	Perceived benefits	0.76		
PB16	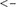	Perceived benefits	0.760		
PB17	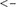	Perceived benefits	0.758		
PR18	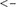	Perceived risk	0.789	0.6107	0.8625
PR19	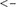	Perceived risk	0.799		
PR20	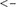	Perceived risk	0.748		
PR21	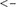	Perceived risk	0.789		
SC22	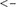	Stability of cooperation	0.919	0.678	0.9363
SC23	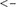	Stability of cooperation	0.815		
SC24	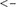	Stability of cooperation	0.778		
SC25	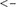	Stability of cooperation	0.806		
SC26	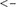	Stability of cooperation	0.797		
SC27	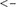	Stability of cooperation	0.786		
SC28	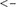	Stability of cooperation	0.854		
T29	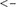	Trust	0.927	0.6544	0.9187
T30	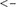	Trust	0.764		
T31	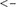	Trust	0.787		
T32	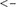	Trust	0.777		
T33	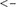	Trust	0.816		
T34	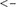	Trust	0.771		

**TABLE 2:** Discriminant validity analysis of the evaluation model of cooperation stability in the agricultural industry chain.

Latent variable	IP	MP	NP	PB	PR	SC	T
IP	0.6819	–	–	–	–	–	–
MP	0.334	0.6093	–	–	–	–	–
NP	0.265	0.261	0.6717	–	–	–	–
PB	0.27	0.259	0.268	0.6073	–	–	–
PR	-0.236	-0.3	-0.229	-0.257	0.6107	–	–
SC	0.292	0.307	0.305	0.296	-0.297	0.678	–
T	0.091	0.048	0.086	0.019	-0.033	0.096	0.6544
AVE square root	0.826	0.781	0.820	0.779	0.781	0.823	0.809

### 4.2. Hypothesis testing

#### 4.2.1. Related analysis

From Table 3, there is a positive correlation between imitative, mandatory, and normative pressures and perceived benefits, stability of cooperation, and trust, while there is a significant negative correlation between perceived risk, cooperation stability, and trust. It was found that perceived risk was negatively correlated with the cooperation stability and trust, which is in line with the hypothesized results.

**TABLE 3:** Means, standard deviations, and correlation coefficients.

Variables	M	SD	IP	MP	NP	PB	PR	SC	T
IP	3.807	0.826	1	–	–	–	–	–	–
MP	3.696	0.823	0.571**	1	–	–	–	–	–
NP	3.710	0.794	0.476**	0.523**	1	–	–	–	–
PB	3.909	0.702	0.463**	0.457**	0.491**	1	–	–	–
PR	2.446	0.873	-0.325**	-0.520**	-0.364**	-0.370**	1	–	–
SC	3.815	0.768	0.490**	0.575**	0.551**	0.510**	-0.425**	1	–
T	3.816	0.741	0.458**	0.512**	0.431**	0.423**	-0.432**	0.479**	1

**Significant correlation at the 0.01 level.

#### 4.2.2. Mediating effect test

In order to test the direct and mediating effects of the model, this study used the Bootstrapping method in PROCESS, an SPSS plug-in designed by Hayes (2017), to conduct a 5,000 times sampling to obtain 95% unbiased corrected confidence intervals. The PROCESS model 80 was selected for testing the composite multiple mediating effects of perceived benefits, perceived risks, and trust as mediators of the relationship between imitative pressure, mandatory pressure, normative pressure, and cooperation stability, as shown in Tables 4–8.

**TABLE 4:** Analysis of the relationship between variables in the composite multiple mediating effect model of imitative pressure and cooperation stability based on regression analysis.

Variables	PB			PR			T			SC		
	B	SE	T	B	SE	t	B	SE	t	β	SE	t
Gender	-0.111	0.080	-1.395	−0.010	0.106	-0.089	−0.073	0.078	-0.925	-0.158	0.076	-2.077
Age	-0.011	0.058	-0.187	−0.042	0.077	-0.546	0.084	0.056	1.488	0.011	0.055	0.206
IP	0.388	0.046	8.457***	−0.347	0.061	-5.686***	0.258	0.051	5.007***	0.210	0.052	4.021***
PB	–	–	–	–	–	–	0.201	0.062	3.252**	0.276	0.061	4.528***
PR	–	–	–	–	–	–	−0.228	0.046	-4.912***	-0.156	0.047	-3.322**
T	–	–	–	–	–	–	–	–	–	0.189	0.059	3.201**
R^2^	0.220			0.107			0.334			0.418		
F	25.499			10.799			26.997			32.032		

**P < 0.01, ***P < 0.001.

**TABLE 5:** Analysis of regression between variables in the composite multiple mediating effect model based on mandatory pressure and cooperation stability.

Variables	PB			PR			T			SC		
	β	SE	t	β	SE	t	β	SE	t	β	SE	t
Gender	-0.129	0.080	-1.621	−0.006	0.096	-0.066	−0.082	0.078	-1.051	-0.169	0.074	-2.270
Age	-0.034	0.058	-0.585	−0.034	0.069	-0.499	0.072	0.056	1.281	0.006	0.053	0.114
MP	0.386	0.046	8.398***	−0.553	0.055	-10.029***	0.290	0.056	5.227***	0.308	0.055	5.579***
PB	–	–	–	–	–	–	0.215	0.060	3.5785***	0.272	0.058	4.648***
PR	–	–	–	–	–	–	−0.160	0.050	-3.2036**	-0.081	0.049	-1.678
T	–	–	–	–	–	–	–	–	–	0.161	0.058	2.7772**
R^2^	0.218			0.271			0.339			0.447		
F	25.162			33.558			27.615			36.067		

**P < 0.01, ***P < 0.001.

**TABLE 6:** Analysis of the correlation between variables in the composite multiple mediating effect model of normative pressure and cooperation stability.

Variables	PB			PR			T			SC		
	β	SE	t	β	SE	t	β	SE	t	β	SE	t
Gender	-0.097	0.079	-1.234	−0.025	0.105	-0.241	−0.069	0.080	-0.863	-0.149	0.075	-2.003
Age	-0.002	0.057	-0.041	−0.052	0.076	-0.692	0.082	0.058	1.431	0.022	0.054	0.407
NP	0.430	0.047	9.119***	−0.406	0.063	-6.471***	0.220	0.056	3.928***	0.294	0.054	5.485***
PB	–	–	–	–	–	–	0.218	0.063	3.447**	0.239	0.060	3.967***
PR	–	–	–	–	–	–	−0.229	0.048	-4.817***	-0.136	0.046	-2.936**
T	–	–	–	–	–	–	–	–	–	0.186	0.057	3.279**
R^2^	0.246			0.134			0.312			0.445		
F	29.439			13.982			24.347			35.786		

**P < 0.01, ***P < 0.001.

**TABLE 7:** Bootstrap analysis of the relationship between imitative pressure and cooperation stability.

	Effect	Boot SE	Boot LLCI	Boot UlCI	Effectiveness ratio
Total effect	0.450	0.049	0.353	0.546	100.00%
Direct effect	0.210	0.052	0.107	0.313	46.67%
Total indirect effect	0.240	0.053	0.142	0.344	53.33%
IP→PB→SC	0.107	0.037	0.041	0.181	23.83%
IP→PR→SC	0.054	0.025	0.011	0.110	12.03%
IP→T→SC	0.049	0.031	0.001	0.119	10.85%
IP→PB→T→SC	0.015	0.011	0.000	0.041	3.27%
IP→PR→T→SC	0.015	0.009	0.0003	0.036	3.33%

**TABLE 8:** Bootstrap analysis of the mediating effects of mandatory pressure and stability of cooperative relationships.

	Effect	Boot SE	Boot LLCI	Boot UlCI	Effectiveness ratio
Total effect	0.532	0.046	0.442	0.623	100.00%
Direct effect	0.308	0.055	0.200	0.417	57.94%
Total indirect effect	0.224	0.062	0.116	0.358	42.06%
MP→PB→SC	0.105	0.040	0.041	0.193	19.67%
MP→PR→SC	0.045	0.036	–0.025	0.117	–
MP→T→SC	0.047	0.033	0.000	0.126	8.75%
MP→PB→T→SC	0.013	0.009	–0.0002	0.036	–
MP→PR→T→SC	0.014	0.011	–0.001	0.040	–

(1) Regression analysis results

As shown in Table 4, the effect of imitation pressure on perceived benefits, trust, and cooperation stability was positive (β = 0.388, t = 8.457, *p* < 0.001; β = 0.258, t = 5.007, *p* < 0.001; and β = 0.210, t = 4.021, *p* < 0.001, respectively), and the effect was negative on perceived risk (β = −0.347, t = −5.686, *p* < 0.001). Therefore, hypothesis 1b and hypothesis 1c are supported. In addition, perceived benefits positively affect trust and cooperation stability (β = 0.201, t = 3.252, *p* < 0.01; and β = 0.276, t = 4.528, *p* < 0.001, respectively) and perceived risks negatively influence trust and cooperation stability (β = −0.228, t = −4.912, *p* < 0.001; and β = −0.156 t = −3.322, *p* < 0.01, respectively). Trust positively influences the cooperation stability (β = 0.189, t = 3.201, *p* < 0.01).

As shown in Table 5, mandatory pressure positively affects perceived benefits, trust, and stability of cooperative relationships (β = 0.386, t = 8.398, *p* < 0.001; β = 0.290, t = 5.227, *p* < 0.001; and β = 0.308, t = 5.579, *p* < 0.001, respectively) and negatively affects perceived risk (β = −0.553, t = −10.029, *p* < 0.001). Therefore, hypothesis 2b and hypothesis 2c are supported. Perceived benefits positively influence trust and cooperation stability (β = 0.215, t = 3.5785, *p* < 0.001; and β = 0.272, t = 4.648, *p* < 0.001, respectively), and perceived risk negatively influences trust (β = −0.160, t = −3.2036, *p* < 0.01). However, the effect of perceived risk on cooperation stability is not significant. Trust positively affects cooperation stability (β = 0.161, t = 2.7772, *p* < 0.01).

As shown in Table 6, normative pressure positively affects perceived benefits, trust, and cooperation stability (β = 0.430, t = 9.119, *p* < 0.001; β = 0.220, t = 3.928, *p* < 0.001; and β = 0.294, t = 5.485, *p* < 0.001, respectively) and negatively affects perceived risk (β = −0.406, t = −6.471, *p* < 0.001). Therefore, hypothesis 3b and hypothesis 3c are supported. Perceived benefits positively influence trust and cooperation stability (β = 0.218, t = 3.447, *p* < 0.01; and β = 0.239, t = 3.967, *p* < 0.001, respectively), and perceived risks negatively influence trust and cooperation stability (β = −0.229, t = −4.817, *p* < 0.001; and β = −0.136 t = −2.936, *p* < 0.01, respectively). Trust positively influences cooperation stability (β = 0.186, t = 3.279, *p* < 0.01).

From Tables 4–6, hypotheses 4, 5a, and 5b can all be verified to be true. That is, trust contributes positively to cooperation stability in the agricultural industry chain, perceived benefits have a significant and positive effect on trust, and perceived risk has a significant and negative effect on trust.

(2) Mediating effect analysis results

**FIGURE 2:**
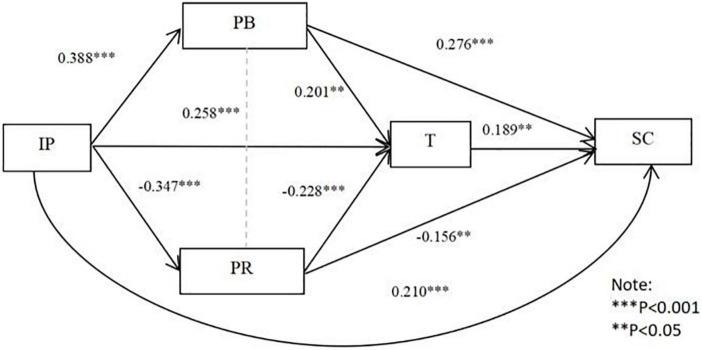
Imitative pressure and stability of cooperation: A composite multiple intermediaries of perceived benefits, perceived risk, and trust.

From Table 7 and Figure 2, imitative pressure has a significant direct effect on cooperation stability (95% CI = 0.107, 0.313), accounting for 46.67% of the total effect, and hypothesis 1a is supported. Perceived benefits, perceived risk, and trust are significant mediators of imitative pressure and cooperation stability [95% CI = (0.041, 0.181), 95% CI = (0.011, 0.110); 95% CI = (0.001, 0.119)], accounting for 23.83, 12.03, and 10.85% to the total effect, respectively. In addition, the chain mediation of perceived benefit and trust in imitative pressure and cooperation stability is significant [95% CI = (0.000, 0.041)], accounting for 3.27% of the total effect, and the chain mediation of perceived risk and trust in imitative pressure and cooperation stability is also significant [95% CI = (0.0003, 0.036)], accounting for approximately 3.33% of the total effect. Therefore, the composite multiple mediating effect model of perceived benefits, perceived risk, and trust is valid, and hypothesis 6a is supported.

**FIGURE 3:**
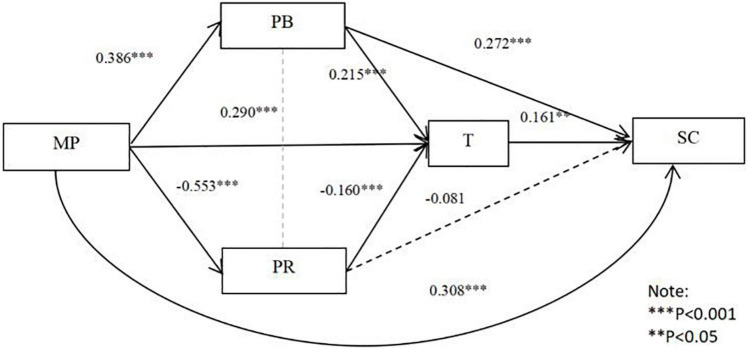
Mandatory pressure and stability of cooperation: Conforming multiple mediators of perceived benefits, perceived risk, and trust.

From Table 8 and Figure 3, the direct effect of mandatory pressure on the stability of cooperation is significant [95% CI = (0.200, 0.417)], accounting for 46.67% of the total effect, and hypothesis 2a is supported. A significant mediating effect is found between perceived benefits and trust in mandatory pressure and cooperation stability of [95% CI = (0.041, 0.193); 95% CI = (0.000, 0.126)], accounting for 19.67% and 8.75% of the total effect, respectively. However, the perceived risk does not have a significant contribution in mediating the relationship between mandatory pressure and cooperation stability, and hypothesis 2c is not supported. The chain mediation of perceived benefits and trust in mandatory pressure and stability of cooperation is not significant [95% CI = (0.000, 0.041)]; perceived risk and trust are also not significant chain mediators in mandatory pressure and cooperation stability [95% CI = (0.0003, 0.036)], thus hypothesis 6b is not supported. Nevertheless, it is equally important to consider the role of perceived benefits and trust in the relationship between mandatory pressure and cooperation stability.

**FIGURE 4:**
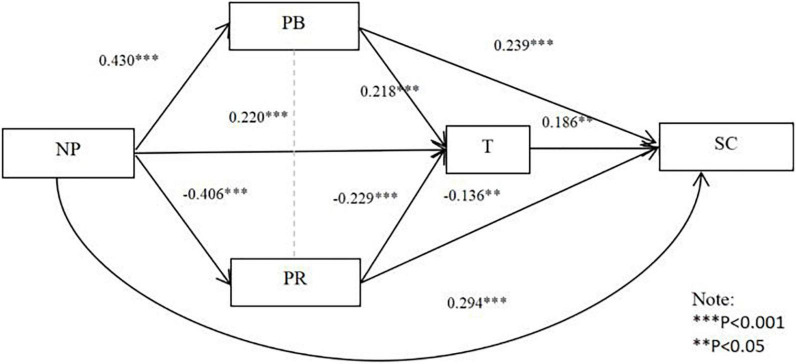
Normative pressure and stability of cooperation: Conforming multiple mediators of perceived benefits, perceived risk, and trust.

From Table 9 and Figure 4, normative pressure has a significant direct effect on cooperation stability [95% CI = (0.189, 0.400)], which accounts for 55.76% of the total effect, and hypothesis 3a is supported. The mediating effects of perceived benefits, perceived risks, and trust between normative pressure and cooperation stability are significant [95% CI = (0.020, 0.195); 95% CI = (0.010, 0.117); 95% CI = (0.002, 0.105)], accounting for 19.47, 10.42, and 7.77% of the total effect, respectively. In addition, a significant chain mediation is found between perceived benefits and trust in normative pressure and cooperation stability [95% CI = (0.001, 0.041)], accounting for 3.31% of the total effect. The chain mediation of perceived risks and trust in normative pressure is also significant (95% CI = (0.001, 0.042), accounting for 3.28% of the total effect. Therefore, the composite multiple mediating effect model of perceived benefits, perceived risk, and trust is valid, and hypothesis 6c is supported.

**TABLE 9:** Bootstrap analysis of whether normative pressure has a mediating effect on the stability of cooperative relationships.

	Effect	Boot SE	Boot LLCI	Boot UlCI	Effectiveness ratio
Total effect	0.528	0.049	0.432	0.625	100.00%
Direct effect	0.294	0.054	0.189	0.400	55.76%
Total indirect effect	0.234	0.055	0.133	0.350	44.24%
NP→PB→SC	0.103	0.045	0.020	0.195	19.47%
NP→PR→SC	0.055	0.027	0.010	0.117	10.42%
NP→T→SC	0.041	0.027	0.002	0.105	7.77%
NP→PB→T→SC	0.018	0.010	0.001	0.041	3.31%
NP→PR→T→SC	0.017	0.010	0.001	0.042	3.28%

## 5. Discussions

In this study, we investigated the mechanisms of how institutional pressures influence cooperation stability in the agricultural industry chain. The questionnaire survey method was used to test the research hypotheses in this study, and the results are as follows.

First, institutional pressure can contribute to the stability of cooperation in the agricultural industry chain. Three dimensions of institutional pressure, i.e., imitative pressure, mandatory pressure, and normative pressure, all have positive predictive effects on the stability of cooperation in the agricultural industry chain. It indicates that the existence of cooperative institutions can exert imitative pressure, mandatory pressure, and normative pressure on agricultural industry chain operators, prompting agricultural industry chain operators to abide by cooperative rules and establish cooperative norms and atmosphere, and help maintain stable cooperative relationships and development trends in agricultural industry chains. This is consistent with the findings of previous studies by Peng et al. (2018) and Jiao et al. (2021b) on the impact of institutional pressure on behavioral intention.

Second, perceived benefits, perceived risks, and trust play composite multiple mediating roles in imitative pressure, normative pressure, and cooperation stability in the agricultural industry chain. However, the composite multiple mediating effects are not significant in the relationship between mandatory pressure and cooperative relationships stability. Specifically, in agricultural industry chains, imitation pressure and normative pressure can influence the stability of cooperation by influencing perceived benefits and perceived risks. Furthermore, through perceived benefits and perceived risks, agricultural industry chain operators can also gain trust in cooperative partners, and finally, realize stable cooperation based on trust. However, mandatory pressure can only affect the stability of cooperation by influencing the perceived benefits or trust level of agricultural industry chain business subjects rather than influencing the perceived risks. It is even less able to leverage perceived value to further influence trust. This indicates that when agricultural industry chain operators face pressure from the cooperative system, they will pay more attention to the imitative pressure and normative pressure from the system, and these two pressures will influence the value judgment and trust in stable cooperation. In contrast, the mandatory pressure from the system does not appear to be effective.

This is partially consistent with the findings of Lin et al. (2019), which show that the strong pressure from strong agents does not promote the stability of cooperative relationships in agricultural industry chains, and the facilitative effect of mandatory pressure on cooperation will not be realized in the presence of risk shocks unless the benefits of cooperation can be clarified, because the mandatory pressure doesn’t facilitate the risk perception assessment. In addition, mandatory pressure occurs through formal or informal pressure exerted by strong subjects on weak subjects in the field, and weak subjects are in a position of passively accepting institutional constraints. In this situation, the pursuit of mandatory pressure does not lead to better cooperation, but rather leads to rebellion and even mistrust, which in turn threatens the stability of cooperative relationships in the agricultural industry chain.

Finally, perceived benefits and perceived risks can antecedently affect the trust of agricultural industry chain operators. Previous studies have disagreed with the causal relationship between trust and perceived benefits and perceived risks, with some scholars arguing that the two are juxtaposed (Kim and Prabhakar, 2004) and others arguing that trust is an antecedent factor (Wang and Yan, 2022; Yan, 2022). As these results suggest, institutional pressure affects both the perceived benefits and perceived risks of agricultural industry chain operators, which ultimately affects their behavior and trust. And this result is related to the specificity of the agricultural industry chain. This is because the development of agriculture is heavily dependent on the geographical environment. In most cases, agricultural operators in the same region have kinship or other acquaintance economic relationships. These relationships have laid the foundations of trust in the cooperative business of the agricultural industry chain. Previously, before formal institutions or relationships are formed, informal institutions or relationships based on relational trust have contributed to and sustained cooperation in the agricultural industry chain to some extent. When institutional pressure emerges, agricultural industry chain operators form perceived benefits and perceived risks based on the system’s education and guidance for cooperative management, as well as their judgment of the current and future benefits of cooperative management. Moreover, perceived benefits and risks emerge before further enhancing or weakening the existing trust relationship, which will ultimately affect the stability of cooperative relationships in the agricultural industry chain. Therefore, the integration of the agricultural industry chain is the integration of market logic as the factor allocation relationship and the economic influence of acquaintances on business behavior.

## 6. Research conclusion and management implications

This study examined the effects of institutional pressure (imitative pressure, mandatory pressure, and normative pressure) on the stability of the agricultural industry chain through a mediated effects test. The results showed that imitative pressure and normative pressure can directly influence the stability of cooperative relationships in the agricultural industry chain, and also indirectly influence the stability of cooperative relationships in the agricultural industry chain through a composite multiple mediating effects model of perceived benefits, perceived risks, and trust. However, only mandatory pressure and the separate mediating effects of perceived benefits and trust were as significant as imitative pressure and normative pressure in affecting the stability of cooperative relationships through the composite multiple mediating effects.

### 6.1. Theoretical implications

Firstly, this study explores institutions’ effect on the stability of cooperative relationships in the agricultural industry chain based on the institutional theory, expanding the application of institutional theory. Institutional theory has been used to analyze the institutional activities of individuals, organizations, or inter-organizations. However, the agricultural industry chain contains both organizations and individuals, and there are both cooperations between individuals and organizations and inter-organizations, which are the uniqueness of the agricultural industry chain. Institutional theory can be expanded in depth and breadth by applying it to the complex systemic context of the agricultural industry chain.

Secondly, the mechanisms of the stability of cooperative relationships in the agricultural industry chain are examined in more detail. Previous studies mainly investigated industrial chain cooperation from the perspective of the game of interests. Although institutional factors are also involved, there has been no in-depth analysis of internal mechanisms of the institutional influence on the group and individual behavior, as if there is a “black box” of influence mechanisms at work. In this study, institutional pressure was divided into imitative pressure, mandatory pressure, and normative pressure, and the influence of different pressure structures on cooperative relationships was examined separately. Meanwhile, considering perceived risks, perceived benefits, and trust factors, the inner mechanism of stable cooperation between institutions was clarified.

Third, on the basis of the agricultural industry chain and the scenario of industry chain cooperation, the relationship between perceived benefits, perceived risks, and trust are empirically examined. In previous studies, different conclusions have been drawn regarding the relationship between perceived benefits, perceived risks, and trust. In this study, the perception of benefits and perceived risk are the antecedents of trust in the context of the agricultural industry chain, which provides a basis for future research on the trust psychology of chain operators.

### 6.2. Management implications

The stability of cooperative relationships in the agricultural industry chain is conducive to the stability and increase of agricultural production, the stable income growth for farmers, and the stability and peace in the countryside. This study analyses the mechanisms underlying the stability of cooperative relationships in agricultural industry chains, which can help the government and other management entities to take targeted measures to deal with instability in the development of agricultural industry chains. It can also help governments to reduce the cost of policy implementation and improve the lag in policy effects.

First, it is found that institutional pressure has significant effects on the stability of cooperation in the agricultural industry chain from three perspectives: imitative pressure, mandatory pressure, and normative pressure. Therefore, the leading operators of agricultural industry chains and the government can take measures to enhance the stability of agricultural industry chains, strengthen guidance and publicity, create benchmarks and role models, emphasize the importance of stable cooperation, and increase imitative pressure within the industry chain. The government should help the agricultural industry chain to establish a reasonable and legal cooperation system, improve the contracting system and cooperation norms, and provide legal and regulatory endorsements for the cooperation system of the cooperative operations of the agricultural industry chain, which can ensure the stable cooperation and assist the leading enterprises to build their authority and enhance mandatory pressure within the chain. Efforts should be made to build a community of destiny within the agricultural industry chain, strengthen education and guidance, foster a behavioral logic of stable and cooperative concerted action within the chain, and complement various forms of institutional regulation to promote mutual mandatory and normative pressures.

Second, the community of destiny of the agricultural industry chain should be established by relying on the village or county. From the study, we found that trust has a positive influence on the stability of cooperation in agricultural industry chains. Perceived value and trust play composite multiple mediating roles between institutional pressure and cooperation stability. Therefore, relying on the village or county to create an agricultural industry chain community of destiny can promote the integration of rural primary, secondary and tertiary industries and embed a social network of acquaintances of agricultural business subjects into the development of rural industrial economies. For example, integrate local characteristic industrial resources using clan networks, thereby achieving factor integration and agglomeration and ensuring stable and intensive cooperation. On the one hand, based on clan networks and acquaintance societies, the trust relationship between industrial chain business subjects is better than that among non-acquaintance networks, and the intermediary role of trust is maximized. On the other hand, in addition to promoting the development of industrial value, industry chain cooperation can also enhance the level of social interaction among business subjects and increase the perceived benefits of stable industry chain cooperation due to the accumulation of social capital, which goes beyond the value of the benefits. Moreover, since the industrial chain operating subjects are in a social network of acquaintances, when a risk shock occurs, the perceived risk of operating subjects is lower than under a simple contractual arrangement.

## Data availability statement

The raw data supporting the conclusions of this article will be made available by the authors, without undue reservation.

## Author contributions

R-RZ and Q-HC wrote the draft of the manuscript. R-RZ and QW contributed to the data curation and analysis. Q-HC and YT contributed to the manuscript revision. All authors contributed to the article and approved the submitted version.
